# A Practical Treatise on Dental Medicine, Being a Compendium of Medical Science, as Connected with the Study of Dental Surgery

**Published:** 1851-03

**Authors:** 


### BIBLIOGRAPHICAL NOTICES.

*A Practical Treatise on Dental Medicine, being a compendium of Medical Science, as connected with the study of Dental Surgery.* By THOMAS E. BOND, A. M. M. D., Professor of Special Pathology and Therapeutics in the Baltimore College of Dental Surgery. Lindsay & Blakiston, Philadelphia, 1851.

Professor Bond has here produced a work invaluable alike to the physician, the medical student, and the dentist. To speak in terms of the warmest praise and commendation of this volume,—of its matter and manner,—its evidences of learning, and yet of careful adaptation to the wants of the student, is to give but an imperfect estimate of its claims to respect and gratitude from medical men, and especially dentists ; for the author has stepped aside (we had almost said *descended*, with a conscious sense of the injustice dealt out to dentists and dentistry,) from the path of his legitimate duties, and the exalted field of research applicable to his practice as a physician, to prove and explain the important connection existing between a neglected speciality and its parent science. His book is calculated to simplify the history of their mutual dependence, and the task has been performed with such a master pen, as must produce great good t othe representatives of both professions.

The frank physician who reads this volume will necessarily acknowledge with some self-reproach, that he has overlooked important knowledge, and relatively important duties: and the dentist will feel with pride that he has found a friend who appreciates, and sympathizes with, his department of science—one who is competent to teach him valuable truths that govern the principles of his art. The work of Professor Bond is destined, we trust, to strengthen in the minds of professional men, the true affinity subsisting between general pathology and that which is special of the teeth: and in this nearing of one branch to the main tree, may we not hope to perceive also a nearer approach of sympathies between the good and sensible men to be found in the two professions—physician and dentist.

The useful and available knowledge in these 324 pages, from a clear and gifted mind, embraces every fundamental principle applicable to a medico-dental education; and the author, by furnishing this basis for the studies of the young dentist, has done much towards affording that opportunity of good preparation which may result in accomplishing his own just wishes. When speaking of a great surgical operation successfully executed by a distinguished dentist of our country, he says:—"It is the more worthy of notice here, as having been performed by a dentist, who thus furnishes a model of what we would have a dentist to be. Not a mere mechanic, employed to repair the teeth, or, if necessary, to extract them—but an accomplished physician and surgeon, who, while devoting his attention peculiarly to the teeth, is prepared to undertake the treatment of adjacent parts, however formidable and complicated their diseases may be."

This high standard of ability demanded by Professor Bond for the dentist, and of which he justly cites Dr. S. P. Hullihen as an example, may not, however, be readily reached in the large cities, as the professions are now divided; we do not say it as an excuse for ignorance, but as a cause for neglect on the part of the dentist to practice the general surgery of the mouth and surrounding parts—operating for hare-lip—diseases of the palate, tonsils, &c. &c.

For the same reason that medical men often extract teeth and relieve toothach in country practice, where a reliable dentist is not to be had, so the accomplished dentist in remote places, where good surgeons are not accessible, may very properly perform the operations of general surgery—always supposing he has the right given by a diploma of M. D. from a medical school. The physicians of the cities do not meddle with the duties or prerogative of the dentists—and it is but just, as well as for the best interests of the community, that this feeling should be reciprocal.

It may be safely averred, and without invidious feeling, that the general surgeons or medical men of our large cities, would regard such practice by the dentists as encroachments, perhaps impertinent ones, upon their province, and even the successful operations would have no tendency to decrease the jealous assumption of superiority, too common among physicians towards dentists. We all know there are some of them, who, while occupying high rank professionally, are far from being great men in just or generous impulses of mind or heart.

But the fearless justice of Professor Bond is an exception to this rule, and is no less commendable, than his true discrimination; he does not hesitate to give honor where honor is due—even though Mr. Liston and his editor both stand in the way. He has done justice to Dr. Mott, with reference to his being the first who successfully amputated the lower jaw at the articulation; and to Dr. S. P. Hullihen, of Wheeling, Va., for a no less difficult and original operation, which proves him to be, says Dr. Bond, "*one of the best surgical operators in this country.*"

The time will perhaps come when such models will be imitated, and when *general* surgeons will be less unjust and ungenerous to *special* ones, *even though they should be dentists*; then we may realize the blessed state of things proposed for dental education by Professor Bond. But it is not likely to be until after the public, or its sensible portion, ceases to countenance *self-made* dentists—dentists metamorphosed spontaneously from broken down watch-makers, silversmiths, adventurers, or blacksmiths; and when the distinctions between mercantile and professional occupations are widely felt, and made to affect the dentist—when moral character and gentlemanly habits are demanded as much in the performance of operations on the teeth, in the dentist's office, as in the sick room by the bed-side of a suffering patient.

Trading in teeth and tools would seem now to be considered as much the evidence of a surgeon dentist, as chartering or freighting a ship, the sign of a merchant; and we cannot sufficiently express our grateful and respectful approbation of the correct and far reaching sentiment conveyed by Professor Bond in the preface to the book we have attempted to notice.

He says, "The Baltimore College of Dental Surgery was organized with the design of teaching dentistry as a regular branch of medicine, in which relation only, *it can be regarded as a scientific pursuit, and the practice of it esteemed a profession.*"

We earnestly desire that the *design* may result in establishing the important and true distinctions the Professor had in view, but we have been compelled to doubt, whether the establishment of such separate institution, for the *specialities* of medical science would not ultimately affect unfavourably the *only* relation in which dentistry "*can* be regarded as a *scientific pursuit*, and the *practice of it a profession.*"

But we will not be tempted here to enter upon the discussion of the tendency of dental colleges—a fruitful and delicate question, about which we have thought deeply and disinterestedly. We only desire now to approve, and recommend warmly and sincerely, the valuable work given to the profession by Dr. Bond, and we are confessedly unable to do it all the justice, with our pen, which we feel that it deserves.

